# Changes in Bacterial Composition of Zucchini Flowers Exposed to Refrigeration Temperatures

**DOI:** 10.1100/2012/127805

**Published:** 2012-04-01

**Authors:** F. Baruzzi, M. Cefola, A. Carito, S. Vanadia, N. Calabrese

**Affiliations:** Institute of Sciences of Food Production, National Research Council of Italy (CNR-ISPA), Via. G. Amendola 122/o, 70126 Bari, Italy

## Abstract

Microbial spoilage is one of the main factors affecting the quality of fresh fruits and vegetables, leading to off-flavor, fermented aroma, and tissue decay. The knowledge of microbial growth kinetics is essential for estimating a correct risk assessment associated with consuming raw vegetables and better managing the development of spoilage microorganisms. This study shows, for the first time, that only a part of total microbial community, originally present on fresh harvested female zucchini flowers, was able to adapt itself to refrigerated conditions. Through the study of microbial growth kinetics it was possible to isolate forty-four strains belonging to twenty-two species of the genera *Acinetobacter, Arthrobacter, Bacillus, Enterobacter, Erwinia, Klebsiella, Pantoea, Pseudoclavibacter, Pseudomonas, Serratia, Staphylococcus,* and *Weissella*, suggesting *Enterobacteriaceae* as potentially responsible for pistil spoilage.

## 1. Introduction

The fresh-like and healthy traits of ready-to-use (RTU) products such as leafy vegetables (lettuce, rocket, spinach, etc.) and fruits (melon, pineapple, apricot, peach, apple, etc.), with a minimal time of preparation before consumption, are characteristics sought by consumers [1–3]. Microflora causing release of off-flavors and tissue decay is well known in both RTU vegetables and fruits [4–9]. The dominating bacterial population during low temperature storage mainly consists of species belonging to the *Pseudomonadaceae *and *Enterobacteriaceae; *besides some species belonging to the lactic acid bacteria and moulds may be present at relatively lower numbers [4, 10–13].

The consumption of zucchini (*Cucurbita pepo* L.) flowers with the immature fruit attached is spread in many countries, specially in the Mediterranean area, but, due to their high perishability, they are in general marketed only locally.

Different authors [14, 15] studied the effects of cold storage on zucchini flowers processed as a new RTU vegetable; these papers showed that the shelf life of flowers was affected either by low storage temperatures and by cultivars. However, no study is reported in literature on the evolution under refrigerated conditions of natural microbial population occurring on female zucchini flowers that, composed by petals and pistil, presents different tissues and nutrient availabilities.

Aim of this work was to gain a better knowledge about the survival of natural microorganisms occurring on female zucchini flowers, potentially involved in vegetable spoilage, in order to lay the basis for the development of a new RTU vegetable.

## 2. Materials and Methods

### 2.1. Microbiological Analyses of Zucchini Flowers

Microbial evaluation on growth kinetics in zucchini female flowers (cv. Aquilone) was carried out in two consecutive years (trial A and B). Clean flowers, with their immature fruit attached, were harvested and immediately subjected to microbiological analyses.

At the same time, zucchini flowers were stored, as harvested, in gas permeable polypropylene open bags at 4°C ± 1°C for 11 days. Three replicate bags of eight zucchini flowers were prepared for each sampling time (4, 8, and 11 days).

Microbial analysis was carried out on about 25 g of petals and pistils transferred aseptically and separately to a stomacher bag, containing 9 parts (w/w) of sterile saline solution (0.9% NaCl) and homogenized for 1 min using a stomacher (BagMixer, Interscience, St Nom, France). Petal and pistil dilutions were plated in triplicate on different agar media. Total mesophilic bacteria (Plate Count Agar, Oxoid S.p.A. Garbagnate, Milano, Italy) and *Enterobacteriaceae* (Violet Red Bile Glucose Agar) were counted after 24 h of growth at 30°C, yeasts and moulds (Potato Dextrose Agar) at 25°C for four days, whereas presumptive lactic acid bacteria (Rogosa agar under anaerobiosis) were enumerated after 48 h of growth at 30°C. In order to prevent yeast growth, 150 mg L^−1^of cycloheximide were added to Rogosa and PCA, while to prevent bacterial growth 150 mg L^−1^of chloramphenicol were added to PDA. Microbial biotyping and species identification were carried out on isolates from the dominant bacterial population.

For each storage time (0, 4, 8, and 11 days), about 10–15 well-isolated colonies were picked up from PCA plates with the highest number of colonies, inoculated in PC broth (Tryptone 5.0 g L^−1^, Yeast extract 2.5, Glucose 1.0 g L^−1^, Oxoid S.p.A. Garbagnate, Milano, Italy), incubated at 30°C for 24 h and frozen at −80°C for further analyses.

### 2.2. Microbial Biotyping and Species Identification

DNA extraction was carried out using one mL of viable cell suspension by using Wizard Genomic DNA Purification kit (Promega Italia Srl, Milano, Italy) following manufacture's instructions. DNA quantity and quality were determined by electrophoresis with known amounts of molecular weight marker (Marker IV, Roche Diagnostics, Milan, Italy) as a standard.

Isolates were submitted to the two-step RAPD analysis for biotyping [16] using the M13 primer (5′-GAGGGTGGCGGTTCT-3′) capable of characterizing bacterial isolates from apples [17]. After electrophoresis run (2% agarose in TBE buffer, QIAGEN, 70 V for 3–5 h), different isolates showing the same electrophoretic pattern were grouped. Isolates were clustered, sequenced, and identified as recently defined by Baruzzi et al. [18].

### 2.3. Evaluation of Spoilage Symptoms

Fruits and flowers were individually scored for spoilage symptoms (petal necrosis or water soaking, wilting, pistil acid rot, browning, and mycelia growth, etc.) by a group of six trained people using a 5 to 1 subjective scale, with 5 = excellent, no spoilage; 4 = very good, minor spoilage; 3 = fair, moderate spoilage; 2 = poor, major spoilage; 1 = inedible. A score of 3 was considered as the limit of marketability and a score of 2 as the limit of edibility [19].

## 3. Results and Discussion

### 3.1. Enumeration of Different Microbial Populations Occurring on Zucchini Flowers

With the aim to identify the microorganisms, which could influence the spoilage, different microbial populations occurring on pistils and petals were evaluated.

Separating microbial counts by pistils or petals, pistil microbial load was always higher than the corresponding petal microbial population by about 1 or 2 logarithmic unit. The greatest differences in microbial populations were found for total mesophilic aerobic bacteria; in particular, for each sampling time and for both experiments, total mesophilic bacteria on pistils were found to be from 10 to 1000 times more than on petals. The differences between pistils and petals for *Enterobacteriaceae* and presumptive lactobacilli were far less marked. The nutrient availability on pistils can explain the higher microbial growth on pistils rather than on petals.

**Figure 1:**
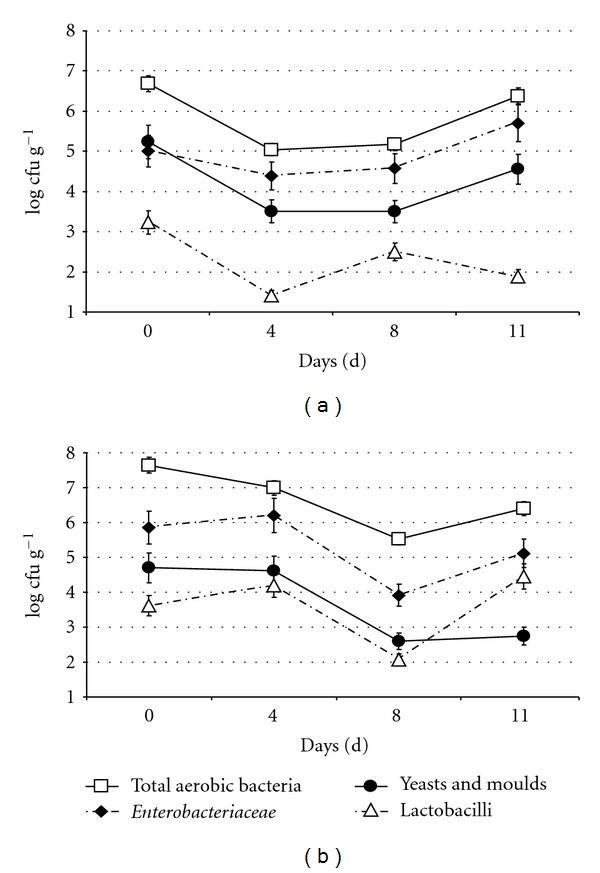
Growth dynamics of microbial populations from zucchini female flower pistils during cold storage at 4°C in trial A and B. Mean value ± standard deviation.

The growth kinetics of total aerobic bacteria, yeasts and moulds, presumptive lactobacilli, and *Enterobacteriaceae *on pistils during the storage at 4°C are shown in Figure 1. The viable cell counts of total aerobic bacteria, yeasts and moulds, and *Enterobacteriaceae *showed a similar behavior for both trials; after an initial reduction, microbial counts remained stable for some days and then increased after eight days in storage, reaching the highest value recorded for flowers in refrigerated conditions at the end of the experiment when spoilage symptoms were detected. In particular, viable cell counts of total aerobic bacteria (Figure 1) decreased from 10 (trial A) to 100 (trial B) times reaching about 6 log cfu g^−1^ in both trials after eleven days of cold storage. The viable *Enterobacteriaceae* counts decreased from 1.0 × 10^5^ to 2.4 × 10^4^cfu g^−1^ in trial A and from 1.6 × 10^6^ to 8.2 × 10^3^ cfu g^−1^ in trial B, increasing to about 5 log cfu g^−1^ at the end of experiment for both trials. Total presumptive lactobacilli, usually known to be related to vegetable spoilage [10], showed counts (Figure 1) that remained relatively low, reaching 2.8 × 10^4^ cfu g^−1^ only after eleven days of cold storage in trial B. Yeasts and moulds decreased 100 times in both trials with final values of 3.6 × 10^4^ cfu g^−1^and 5.6 × 10^2^ cfu g^−1^ in trial A and B, respectively.

We can suppose that, after a selective effect of low temperatures on microflora counted on fresh female zucchini flowers, surviving microorganisms fitted well with the new cold environment, starting to grow. Microbial growth kinetics resulted similarly in both experiments; in particular, total aerobic counts resulted in the dominant microflora, followed by *Enterobacteriaceae*, whereas yeasts and moulds and presumptive lactobacilli remained stable or increased slowly.

Since total mesophilic aerobic bacterial population occurring in pistils was numerically dominant, it was considered, according to Ragaert et al. [2], responsible for female zucchini flower spoilage and then deeper investigated.

### 3.2. Microbial Biotyping and Taxonomic Identification

**Figure 2:**
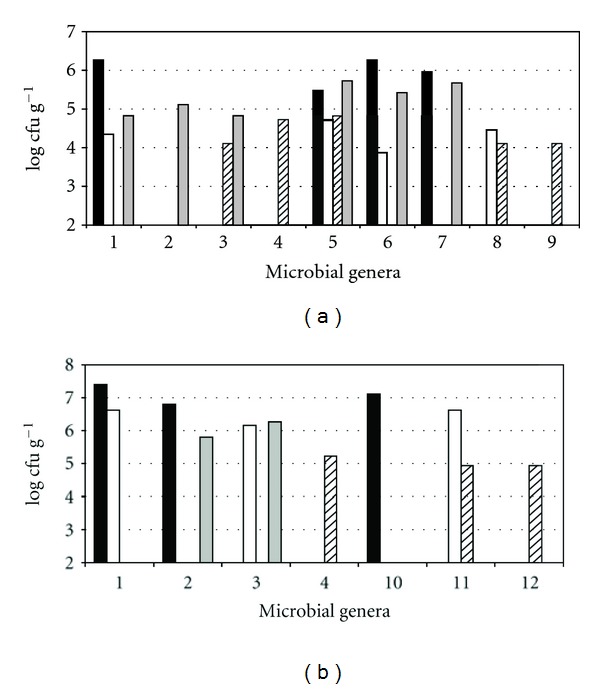
Distribution of bacterial genera and relative total load from zucchini female flower pistils in trial A and trial B after 0 (black bars), 4 (white bars), 8 (diagonal stripes bars), and 11 (grey bars) days of cold storage at 4°C. Bacterial genera are defined as: 1, *Acinetobacter* spp.; 2, *Serratia* spp.; 3, *Klebsiella* spp.; 4, *Pantoea* spp.; 5, *Erwinia* spp.; 6, *Arthrobacter* spp.; 7, *Staphylococcus *spp.; 8, *Weissella *spp.; 9, *Pseudoclavibacter *spp.; 10, *Enterobacter *spp.; 11, *Pseudomonas *spp.; 12, *Bacillus *spp.

Biodiversity of aerobic mesophilic microflora from eight different pistil samples was studied analyzing RAPD-PCR profiles of 118 colonies from PCA plates. In general, every sample showed many different electrophoretic fingerprints, helping to identify the different strains present: clustering 118 colonies gave 44 strains. 16SrDNA analysis showed that in some cases different electrophoretic patterns belonged to the same species, confirming the ability of two-step RAPD analysis to differentiate isolates belonging to the same species. The list of all strains isolated from pistils is presented in Table 1, whereas the distribution and the viable cell load of bacterial genera from pistils during trials A and B are shown in Figure 2. The cluster analysis carried out after RAPD-PCR and 16SrDNA analysis showed wide differences between trial A and B microflora. The trial A showed eight different fingerprints in naturally occurring bacteria on flowers, whereas isolates of trial B were clustered in four groups. During cold storage microbial isolates from trial A were grouped in six, eight, and fourteen RAPD-PCR cluster after four, eight, and eleven days of storage, respectively. The trial B was characterized by a lower level of microbial biodiversity showing six groups of isolates at any sampling time. The unstored flowers microflora was dominated by *Acinetobacter* sp. (Figure 2) in both trials, but the relative microbial load was higher in trial B. Together with this genus, after four days of storage other strains belonging to *Weissella *spp., *Erwinia* spp. and *Arthrobacter* spp. for trial A and *Pseudomonas* spp. and *Klebsiella *spp. for trial B appeared. After eight days, when the early damage symptoms became evident in zucchini flowers in the two experiments, other microbial genera were identified: *Pantoea* spp. in both trials, *Erwinia *spp.,* Pseudoclavibacter *spp. and* Weissella *spp. in trial A or *Pseudomonas *spp. and *Bacillus *spp. isolated from trial B.

At the end of zucchini flowers cold storage, the dominant microflora in trial B were mainly *Klebsiella* and *Serratia *genera. In trial A, in addition to these genera, *Staphylococcus* spp., *Acinetobacter* spp., *Arthrobacter* spp., and *Erwinia* spp. were also detected. The microflora of both trials showed isolates belonging to *Acinetobacter*, *Serratia*, *Klebsiella*, and *Pantoea* genera. Isolates belonging to *Erwinia* spp. were always found in trial A and never in trial B, whereas, differently from trial A, no *Staphylococcus* strain was isolated from trial B samples.

Differences in microflora composition between trials could be also due to different environmental conditions during the growth of the zucchini flowers.

The strains isolated from both trials belonged to the following species: *Acinetobacter calcoaceticus*, *Staphylococcus succinus *and* S. xylosus, Arthrobacter nicotianae, Serratia marcescens, Enterobacter aerogenes, Pantoea agglomerans, *and* Klebsiella oxytoca. *The most frequently isolated species was *Acinetobacter calcoaceticus*, present at each storing day in trial A and initially and after four and eight days in trial B.

*Acinetobacter* and *Staphylococcus* presented five strains; the remaining species showed one or two strains in both trials. As concerns strain growth kinetics, some strains (*A. calcoaceticus, E. persicina, S. marcescens, K. oxytoca*, and* P. agglomerans*) were recovered in more than one sample. The species present at the highest concentrations were *A. calcoaceticus* and *Staphylococcus succinus *(trial A) and *A. calcoaceticus*, *S. marcescens*, and *K. oxytoca* (trial B) isolated from fresh flowers in which total aerobic load showed the highest values. The strains with the lowest microbial load were *A. calcoaceticus* and *K. oxytoca* found at the end of storage in trial A and *Pseudomonas *sp., *Pantoea* sp., *Bacillus megaterium*, and *S. marcescens* detected in trial B. Even when it was possible to identify the same strain in more than one sample, no single strain (or single species) was ever identified as being entirely responsible for the spoilage symptoms showed. The microbial isolates identified during the two trials were quite similar, having many common species. The identification of *Pantoea agglomerans*, *Erwinia persicina*, *Serratia marcescens*, and *Enterobacter aerogenes* in all samples confirms the importance of *Enterobacteriaceae* in microbial spoilage of cold stored vegetables, according to previous indications [1, 10, 20].

*A. baumannii* and *A. calcoaceticus* form a phylogenetically complex of which only *A. calcoaceticus *is usually considered a nonpathogen soil-borne species [21]. We retained reliable the identification of *Acinetobacter *strains as belonging to* A. calcoaceticus* species, when the max score of alignment, calculated against sequences present in the Reference Sequence (RefSeq) database of other *Acinetobacter* species, was no higher than the 95% of that calculated for the top sequence match.

The microbial composition of zucchini flowers here reported partially resembles data from Janisiewicz and Buyer [22] relative to microflora associated with nectarine brown rot, that, in addition with strains belonging to other bacterial genus, reported the isolation of *Enterobacter, Erwinia, Pantoea, Pseudomonas, Serratia*, and *Staphylococcus* strains associated over a period of 7 weeks of nectarine development.

The microbial data showed many species and sometimes different strains of a single species (Table 1); since some strains were found both initially and at the end of cold storage, whereas other grew only during storage, it is possible to assume that only a part of total microbial community, originally present on flowers, was adaptable to refrigerated conditions.

The strain kinetic during cold storage highlighted the microbial biodiversity of flowers, underestimated in fresh flowers that usually are not subjected to severe changes in environmental conditions.

The *Acinetobacter* strains are often associated with food spoilage as previously demonstrated for meat, milk, and cheeses [18, 23, 24].

The finding of some potentially pathogenic bacteria—such as *Acinetobacter spp.* [25], *K. oxytoca, Enterobacter aerogenes *[26], and *Serratia* spp. [27, 28]–commonly found on vegetables, does not represent a risk for consumer health, since these vegetables are usually cooked before eating. Notably, in some cases (*Erwinia persicina*, *Enterobacter aerogenes, Klebsiella oxytoca, *and* Serratia marcescens*) the counts for single strains or species, from PCA plates, was significantly higher (more than 10 times) than *Enterobacteriaceae* counted on VRBGA plates. In addition, in both trials the shape of the *Enterobacteriaceae *growth curve appears the same, but with lower values, of total aerobic bacteria curve.

The results of this work seem to indicate that VRBGA is excessively selective for *Enterobacteriaceae* present on the flowers, leading us to underestimate this microbial load. The presence of 1.5 g L^−1^ of bile salts, needed to evaluate the coliforms present in milk and dairy products, could be selective against *Enterobacteriaceae* naturally occurring on vegetables where bile salts are absent. If similar results will be obtained for other vegetables, then the use of VRBGA for evaluating *Enterobacteriaceae* in these foods should be avoided.

### 3.3. Evaluation of Spoilage Symptoms

During both experiments (trial A and trial B), petals and pistils showed different types of spoilage. Generally, petals remained intact but their orange-yellow color lost natural brightness. In addition, petals turgidity reduction (score 3 or less) was observed after eleven days of refrigerated storage. Pistil spoilage occurred quickly just after eight days of cold storage, when about 80% of pistils appeared brown with gelatinous materials on the surface. Even though we did not perform assays direct to differentiate physiological from microbial spoilage, symptoms on petals remember physiological and biochemical tissue disorders caused by storage at low temperature for long time [29, 30], whereas pistil spoilage resembled soft rot caused by pectolytic and macerating enzymes of many plant pathogenic bacteria on different organs and succulent leaves of vegetables [31, 32]. The evaluation of visual symptoms occurring on petals, pistils, and fruits of female zucchini flowers leads us to identify the pistils as the main perishable part limiting the marketability during the cold storage.

## 4. Conclusions

In this work we evaluated, for the first time, the microbial kinetics of natural occurring microflora of zucchini flowers developing under cold storage conditions; the most perishable part of female zucchini flower was represented by pistils, well known to have high amount of free simple sugars, in comparison with petals, with limited nutrient availability. Molecular tools enabled us to isolate forty-four strains from complex microflora developing on pistils suggesting *Enterobacteriaceae* as potentially responsible for pistil spoilage. However, due to the high microbial biodiversity found in all samples of both trials, the joint responsibility of other bacterial genera cannot be excluded.

A parallel result coming from data comparison from strain biotyping, taxonomic identification, and microbial isolation of different media is that the VRBGA media is excessively selective to evaluate *Enterobacteriaceae* from vegetables.

Microbial data from this work could be useful to set tools and technologies able to preserve the most delicate part of zucchini flower, the pistil, against spoiler microorganisms.

## Figures and Tables

**Table 1:** List of strains identified from zucchini pistils harvested in Trial A and B at 0 (sample T0), 4 (sample T4), 8 (sample T8), and 11 (sample T11) days of cold storage.

Trial A			Trial B		

Strain	Taxonomic identification	Isolated in sample	Strain	Taxonomic identification	Isolated in sample
A5	*Acinetobacter* *calcoaceticus *	T0	C35	*Acinetobacter* *calcoaceticus *	T4
A12	*Acinetobacter* *calcoaceticus *	T0, T4	C36	*Acinetobacter* *calcoaceticus *	T4
A13	*Acinetobacter* *calcoaceticus *	T0, T8, T11	C37	*Acinetobacter* *calcoaceticus *	T4
A83	*Acinetobacter sp.*	T11	C5	*Acinetobacter* *calcoaceticus *	T0, T4, T8
A63	*Arthrobacter* *arilaiti *	T11	C3	*Acinetobacter sp. *	T0
A16	*Arthrobacter* *nicotianae *	T0	C86	*Bacillus* *Megaterium *	T8
A35	*Arthrobacter sp.*	T4	C107	*Enterobacter* *aerogenes *	T11
A80	*Arthrobacter sp.*	T0, T11	C20	*Enterobacter* *aerogenes *	T0
A81	*Arthrobacter sp.*	T11	C74	*Enterobacter* *sp. *	T8
A9	*Erwinia* *persicina *	T0, T4	C110	*Klebsiella* *oxytoca *	T11
A46	*Erwinia* *persicina *	T8, T11	C38	*Klebsiella* *oxytoca *	T4, T11
A38	*Erwinia sp.*	T4	C71	*Pantoea* *agglomerans *	T8
A64	*Erwinia sp.*	T8, T11	C73	*Pantoea* *agglomerans *	T8
A56	*Klebsiella* *oxytoca *	T8	C51	*Pseudomonas sp. *	T4, T8
A72	*Klebsiella sp.*	T11	C122	*Serratia* *marcescens *	T11
A42	*Pantoea agglomerans*	T4	C19	*Serratia* *marcescens *	T0
A57	*Pantoea* *agglomerans *	T8			
A41	*Pantoea sp.*	T8			
A48	*Pseudoclavibacter* *helvolus *	T8			
A77	*Serratia sp.*	T11			
A82	*Serratia sp.*	T11			
A65	*Staphylococcus* *saprophyticus *	T11			
A17	*Staphylococcus* *succinus *	T0, T11			
A20	*Staphylococcus* *succinus *	T0			
A67	*Staphylococcus* *succinus *	T11			
A62	*Staphylococcus* *xylosus *	T11			
A26	*Weissella sp.*	T4			
A53	*Weissella* *viridescens *	T8			
